# Benidipine Protects Kidney through Inhibiting ROCK1 Activity and Reducing the Epithelium-Mesenchymal Transdifferentiation in Type 1 Diabetic Rats

**DOI:** 10.1155/2013/174526

**Published:** 2013-12-01

**Authors:** Ganlin Wu, Meirong Xu, Kui Xu, Yilan Hu

**Affiliations:** ^1^Hubei Province Key Laboratory on Cardiovascular, Cerebrovascular, and Metabolic Disorders, Hubei University of Science and Technology, Xianning 437100, China; ^2^Department of Medicine, Clinic Medical College of Hubei University of Science and Technology, Xianning 437100, China; ^3^Department of Medicine, The Second Affiliated Hospital of Hubei University of Science and Technology, Xianning 437100, China; ^4^Department of Immunology, Wuhan University of Science and Technology, Wuhan 430081, China

## Abstract

We investigated the protective effect of benidipine, by testing the changes of the activity of Rho kinase and transdifferentiation of renal tubular epithelium cells *in vivo*. Wistar rats were randomly divided into two groups: normal (N) and diabetes. STZ were used to make the rats type 1 diabetic and were randomly assigned as diabetes without treatment (D), diabetes treated with benidipine (B), and diabetes treated with fasudil (F) and treated for 3 months. Immunohistochemistry and western blotting were for protein expressions of ROCK1, *α*-SMA, and E-cadherin and real-time PCR for the mRNA quantification of ROCK1. Compared with N group, D group had significant proliferation of glomerular mesangial matrix, increased cell number, thickened basement membrane, widely infiltrated by inflammatory cells and fibrosis in the renal interstitial, and dilated tubular. Those presentations in F and B groups were milder. Compared with N group, D group showed elevated MYPT1 phosphorylation, increased expression of ROCK1, *α*-SMA protein, and ROCK1 mRNA and decreased expression of E-cadherin protein. B group showed attenuated MYPT1 phosphorylation, decreased ROCK1, *α*-SMA protein, and ROCK1 mRNA expression and increased expression of E-cadherin protein. In conclusion, benidipine reduces the epithelium-mesenchymal transdifferentiation and renal interstitial fibrosis in diabetic kidney by inhibiting ROCK1 activity.

## 1. Introduction

Benidipine is a triple calcium channel blocker, simultaneously blocking L, T, and N type channels. It is reported that the effect on T channel is stronger than that on L channel [1], making it a great potential protection for kidney. A number of studies explored the Rho signaling pathway, renal interstitial fibrosis, and tubular epithelium cell transdifferentiation (EMT) [2–4]. The blocking of T calcium channel (TCC) was reported to inhibit the activity of Rho kinase [5], and this is essential in podocyte effacement in immune complex-mediated glomerular disease and other kidney injuries [6]. Furthermore, under cellular stress, Rho kinase activation results in cytoskeletal rearrangement, stress fiber formation, and loss of cellular integrity and function [7]. Rho kinase inhibition prevented these changes and enhanced process formation [8]. These suggested that blocking T channel may have a protective effect on diabetic kidney and reduce epithelium-mesenchymal transdifferentiation and fibrosis via inhibiting ROCK1 (Rho kinase 1) activity.

It was suggested that fasudil, a Rho kinase inhibitor, may attenuate EMT through reduced activation of RhoA/ROCK signaling and be a renoprotective agent for the treatment of DN [9].

Based on that, in this study, we proposed that by inhibiting Rho kinase activity, benidipine reduces epithelium-mesenchymal transdifferentiation and protects kidney in rats with type 1 diabetes (T1DM). By treating type 1 diabetic rats with benidipine and using Rho kinase inhibitor fasudil as positive control, we studied the effects of benidipine on the activity of Rho kinase and EMT in diabetic nephropathy *in vivo*.

## 2. Materials and Methods

### 2.1. Materials

Eight-week old male Wistar rats weighed at 180–200 g (SPF class) were supplied by The Center for Animal Experiment of Wuhan University (Produce Permission no. SCXK (Yu) 2003-0004, Environment Permission no. SYXK (Yu) 2004-0027). Rabbit antibody p-MYPT1 (p853) and E-cadherin antibody were purchased from Bioworld Technology, USA, ROCK1 antibody was purchased from Santa Cruz, USA, rabbit antibody *α*-SMA from Sigma, USA, secondary antibody for internal control protein from Santa Cruz, USA, enzyme-labeling secondary antibody from Sigma USA, Streptozocin (STZ) from Sigma USA, hydrochloride fasudil injection from Tianjin Hongri Pharmaceutical Inc. (lot: 070525), Benidipine from Japanese Kyowa Hakko Kogyo Co., Ltd. (lot: 119AFI), anti-rabbit/rat universal immunohistochemistry kit from Denmark (DAKO), protein extraction buffer from Shanghai Xinghan (DBI), real-time PCR Master Mix from Japanese TOYOBO Biotech, real-time fluorescence PCR equipment from BioRad USA, and the analysis software for fluorescence quantitation was purchased from icycler (version 3.1.7050).

### 2.2. Rat Model Preparation

Fifty-four SPF male Wistar rats were fed with normal chow diet, had free access to water, with room temperature of 20~25°C and relative humidity of 40%~70%, and were in the 12 h light-dark cycle. The rats were randomly assigned into normal group (*n* = 8) and diabetic model group (*n* = 46). After a one-week adaption, the model group was injected intraperitoneally with a single dose of streptozocin (STZ) 60 mg/kg (dissolved in 10 mmol/L citrate buffer, pH 4.5), after a 12-hour fasting. Seventy-two hours after the injection, blood glucose was tested with the samples from tail vein for 3 consecutive days. The criteria for diabetic models were as follows: nonfasting blood glucose is ≥11.1 mmol/L (all was ≥16.7 mmol/L in this study), urine output exceeds the controls over 50%, and urine glucose is strongly positive. During the procedure, 3 rats died and 6 did not meet the criteria. Thirty-seven diabetic rats were randomly assigned into three groups: diabetic without treatment (D, *n* = 13), diabetic treated with fasudil (F, *n* = 12), and diabetic treated with benidipine (B, *n* = 12). Fasudil was injected intraperitoneally with 10 mg/kg/d; benidipine was dissolved in 0.3% carboxymethyl cellulose solution and given via gastric tubing with 3 mg/kg/d. Rats in normal group were injected with citrate buffer. After three months, 8 rats in N group, 9 in D group, 9 in F group, and 8 in B group survived and were sacrificed accordingly.

### 2.3. Samples Collection

One day prior to the sacrifice, 24-hour urine was collected in metabolic chamber. On the same day of sacrifice, tail artery blood pressure was measured with noninvasive blood pressure meter and blood samples were collected. After rinsing with normal saline, some of the kidney tissues were fixed with 10% neutral formalin, embedded with paraffin, made into 3 *μ*m slides, and stained with HE for pathological analysis. The rest of the kidney tissues were stored at −70°C.

### 2.4. The Testing of Biochemistry Parameters

Twenty-four-hour urine protein quantification was measured with sulfosalicylic acid method; serum creatinine (Scr) was tested with picric acid method; blood glucose was tested with glucose oxidase method; and NAG activity was measured with colorimetry as described previously [10].

### 2.5. Immunohistochemistry

Deparaffin the slides routinely, heat repair with microwave, incubate in 3% peroxide at room temperature for 15 minutes, and rinse with PBS (pH 7.4) for three times, 5 minutes for each. Add rabbit antibody p-MYPT1 (1 : 50), *α*-SMA (1 : 50), and E-cadherin (1 : 100) antibody, respectively, and incubate at 4°C overnight. Incubate with horseradish peroxidase-labeled ChemMateTMEnVision secondary antibody at room temperature for 45 minutes, detect with DAB, repeat staining with HE, dehydrate, and seal the film with transparent plastic membrane.

### 2.6. Western Blotting

The total proteins of kidney tissues were extracted with total protein extraction kit. The protein concentration was analyzed with UV spectrophotometry at 260 nm wavelength. Thirty *μ*g of total protein was loaded for SDS-PAGE electrophoresis, then was transferred to nitrocellulose membrane, and then was observed with ponceau S staining. Block with TTBS containing 5% fat-free milk at 4°C for 2 hours. Wash with PBS, then add rabbit anti-rat p-MYPT1 (1 : 1000), ROCK1 (1 : 400), *α*-SMA (1 : 400), E-cadherin (1 : 1000), and *β*-actin (1 : 1000), respectively, and incubate overnight. And then incubate with 1 : 2000 HRP-labeled goat anti-rabbit IgG. Detect with chromogenic agent and expose the film. Scan the image and analyze absorbance with computer software.

### 2.7. Real-Time PCR

Take the kidney cortex tissue 0.1 g from each rat, extract total RNA with TRIzol, and remove genomic DNA with DNase I. Reverse RNA and obtain cDNA. The fluorescence PCR quantification of cDNA was performed with SYBR Green, with triplets for each sample per protocol. The total volume of each reaction was 30 *μ*L, with the following condition: 95°C for 3 min for predenature, then 95°C for 20 s, 60°C for 20 s, and 72°C for 30 s and repeat for 35 cycles, and then 72°C for 5 min. Make a standard curve under 55°C–95°C and save its cycle threshold (CT). Take the CT ratio of each sample to internal control as the relative value of the gene expression of this sample. The primers for ROCK1 and *β*-actin were shown in Table 1.

### 2.8. Statistical Analysis

SPSS (version14) was used for data analysis. Normal distributed quantitative variables were presented as mean ± SD. Comparison among groups was performed with ANOVA, SNK, and LSD tests. Abnormally distributed variables were log-transformed into normal distributed variables and then analyzed thereafter; data were presented with median. *P* < 0.05 was regarded as statistically significant.

## 3. Results

### 3.1. The Changes of 24-Hour Urine Protein (UTP/24 h), Urine N-Acetyl-*β*-glucosaminase (NAG) Activity, Creatinine Clearance (Ccr), Serum Creatinine (Scr), Blood Pressure, and Glucose in Each Group Rats

As shown in Table 2, at 12 weeks, compared with N group, D group had elevated 24-hour urine protein, NAG activity, Scr (*P* < 0.05), and decreased Ccr (*P* < 0.05). Compared with D group, F group had decreased 24-hour urine protein, NAG activity, and Scr (*P* < 0.05). There was no significant difference between F and B groups. There was no significant difference for blood pressure or glucose among groups, as shown in Table 3.

### 3.2. The Pathological Changes of Kidney in Each Group

**Figure 1:**
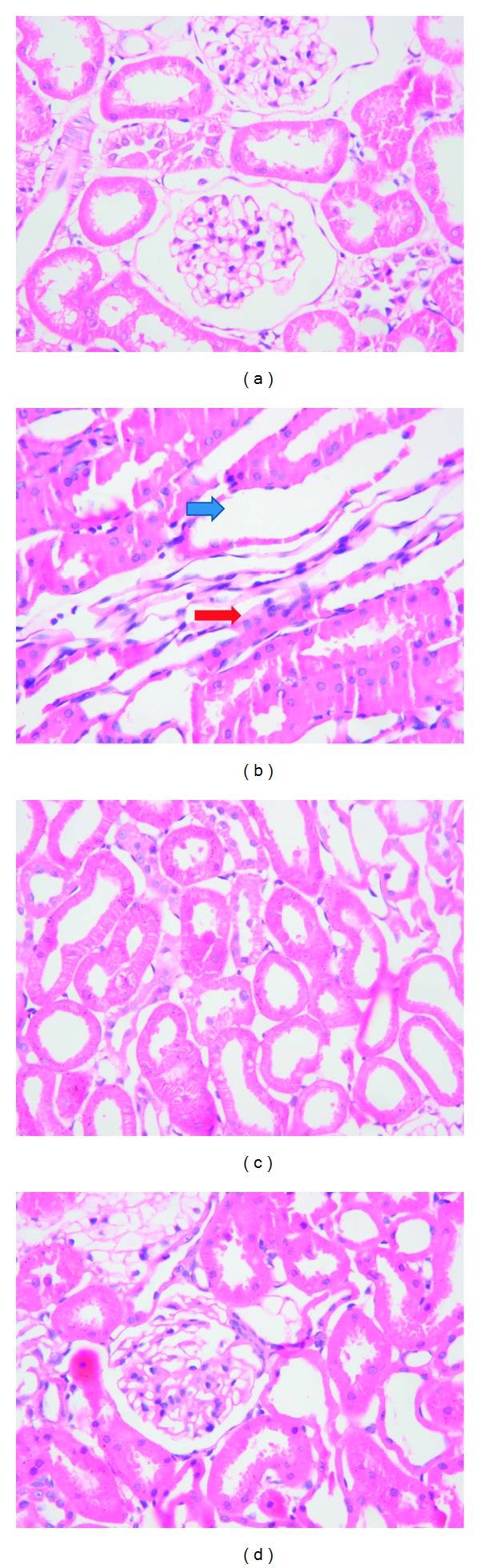
The pathological changes of renal glomerular and interstitial space (×400). (a), (b), (c), and (d) represent N group, D group, F group, and B group, respectively, with HE staining. (a) showed normal tubular; (b) showed tubular dilated and distorted with inflammatory infiltration and fibrosis in renal interstitial space; (c) and (d) showed that the distorted tubular with inflammatory infiltration and fibrosis in renal interstitial were significantly reduced. Blue arrow: tubular dilation; red arrow: inflammatory cell infiltration.

Compared with N and F groups, D group had significant expanded glomerular mesangial matrix, increased cell number, thickened basement membrane, with multiple inflammatory cells infiltrated in interstitial space, dilated renal tubular, and fibrosis in interstitial space. There were mild proliferation of glomerular mesangial matrix, inflammatory infiltration, tubular dilatation, and fibrosis in F and B groups, as shown in Figure 1.

### 3.3. The Changes of Protein Expressions of ROCK1, *α*-SMA, and E-Cadherin in Renal Cortex of the Rats by Immunohistochemistry

**Figure 2:**
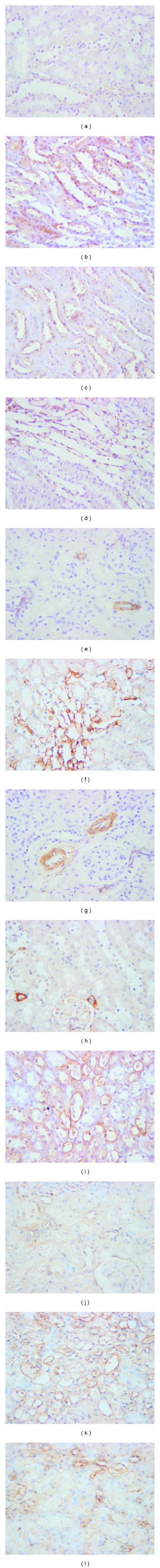
Immunohistochemistry expression (envision ×400). (a), (e), and (i) are for N group; (b), (f), and (j) are for D group; (c), (g), and (k) are for F group; and (d), (h), and (l) are for B group. (a), (b), (c), and (d) are the expression of ROCK1; (e), (f), (g), and (h) are the expression of *α*-SMA; (i), (j), (k), and (l) are the expression of E-cadherin. The brown color in plasma, membrane represented positive expression. (a) showed little expression of ROCK1 in renal tubular epithelium cells; (b) showed widely expressed ROCK1 in renal tubular epithelium cells; (c) and (d) showed the range and degree of staining slighter than those of B group. (e) showed that *α*-SMA is only expressed in the smooth muscle cells of renal small artery; (f) showed that *α*-SMA is expressed in renal tubular epithelium cells; (g) and (h) showed no expression in renal epithelium cells; (i) showed the expression of E-cadherin in renal tubular epithelium membrane; (j) showed no expression; (k) and (l) showed partial expression of E-cadherin on the surface of the cell membrane.

The expression of ROCK1 showed trace in tubular epithelium cells in N group, was enhanced in D group which mainly distributed in dilated renal tubular, and was reduced in F and B groups. *α*-SMA was presented in the smooth muscle cells of renal small artery in N group and visible in epithelium of renal tubular in D group with majority expressed in medullar area but no expression in F or D group. E-Cadherin was mainly presented in the epithelium cells of renal tubular in N group, especially in the cell conjunction area, with partial expression for F and B groups which was enhanced at the cell junction area but no expression on tubular epithelium in D group, as shown in Figure 2.

### 3.4. The Protein Expression of p-MYPT1, ROCK1, *α*-SMA, and E-Cadherin with Western Blotting

**Figure 3:**
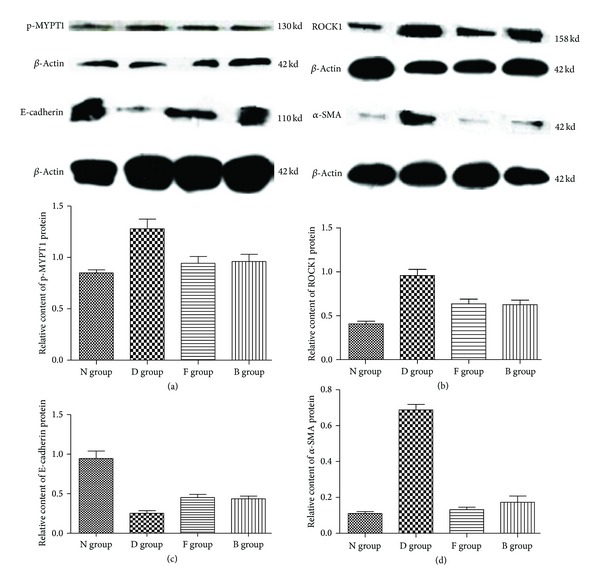
The protein expression changes of p-MYPT1, ROCK1, E-cadherin, and *α*-SMA.

As shown in Figure 3, compared with N group, the rats in D group had enhanced protein expressions of p-MYPT1, ROCK1, and *α*-SMA in renal cortex but reduced E-cadherin. Compared with D group, F group and B group had reduced protein expressions for p-MYPT1, ROCK1, and *α*-SMA and enhanced E-cadherin which was still less than that of the normal group. There was no significant difference between F and B groups, as shown in Figure 3 and Table 4.

### 3.5. Real-Time PCR Showed Changes of mRNA Expression for ROCK1

**Figure 4:**
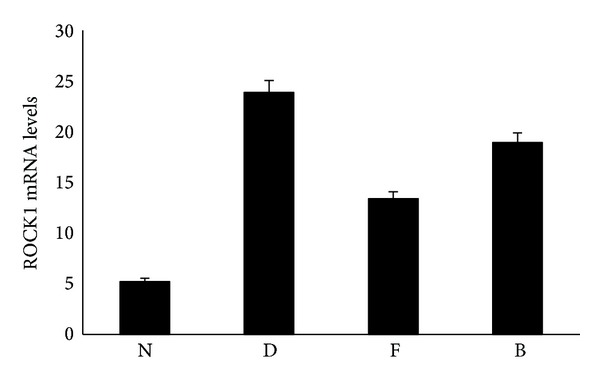
The mRNA expression of ROCK1. Compared with N group, *P* < 0.01; compared with D group, *P* < 0.01.

Compared with N group, mRNA expression of ROCK1 in renal cortex was increased in D group. Compared with D group, there was less mRNA expression of ROCK1 in F and B groups, lower than normal, as shown in Figure 4.

## 4. Discussion

In this study, by treating rats with type 1 diabetic nephropathy with benidipine, a triple channel blocker, and fasudil, a Rho kinase inhibitor, we successfully investigated the effect of benidipine on epithelium-mesenchymal transdifferentiation and its possible mechanism via inhibiting Rho kinase activity. These results were consistent with some previous studies [11].

Rho protein is a small molecular guanylate binding protein. Rho kinase (ROCK) is a widely studied downstream signaling molecule of RhoA. ROCK directly affects myosin light chain (MLC) or indirectly affects the target subunit of myosin phosphatase (MYPT1) and thus increases the phosphorylation of MLC in plasma and controls the attachment, chemoattractant, contraction, and so forth. Phosphorylated MYPT1 level can be used as a marker of ROCK activation. Fasudil is a ROCK-specific inhibitor and inhibits ROCK activity by competitively combining ATP sites of ROCK catalytic domain [12]. ROCKI and ROCKII were both reported. In kidney tissues, ROCKI is the major one presented.

Recent studies revealed that abnormal activation of ROCK signaling pathway played a very important role in the pathophysiology of all kinds of complications of diabetes [13, 14]. Our study confirmed that there was ROCK activation in renal tubular epithelium cells of 12 weeks of diabetic rats, and the effects of ROCK on diabetic renal interstitial space were through ROCK1. Further study found that the protein expression of E-cadherin, the marker protein of renal tubular epithelium cells, was downregulated in diabetic rats, while the protein expression of *α*-SMA, the marker protein of myofibrillar cells, was upregulated, indicating that there was EMT in diabetic nephropathy of rats. Benidipine or fasudil can significantly inhibit NAG activity, reduce urine protein and Scr level, decrease the expression of p-MYPT1, ROCK1, and *α*-SMA, and increase the expression of E-cadherin without affecting blood glucose or pressure. This suggested that benidipine inhibited the activity of ROCK1 and thus partially blocked EMT. Benidipine has protective effect on diabetic nephropathy of rats, independent of its effect of lowering blood pressure.

TCC is a low-voltage activated channel, mainly located in renal efferent arterioles and pacing cells of the heart. Through its strong blocking effects on TCC, benidipine dilates renal afferent and efferent arterioles equally and thus effectively reduces the resistance of renal vessels and intrarenal pressure [15]. It is showed that, besides its effects on adjusting capillary pressure of glomerular, TCC has many nonhemodynamic effects [16–19]. It regulates the activity of NF-k*β* and thus inhibits inflammation, promotes the secretion and release of aldosterone, improves the remodeling of the heart and kidney, and anti-oxygenize and anti-proliferate [20–23]. It is found that in the subremoval kidney models, by inhibiting Rho kinase activity, selective T channel blocker improves renal interstitial fibrosis and EMT. Our study demonstrated that benidipine inhibited Rho kinase activity and thus partially blocked EMT. So far, there is few studies done and the results all supported ours. However, there are limitations of our study; the sample size was rather small, and it is a study in animals instead of human being.

In summary, our study is the first study suggesting that benidipine protects kidney in rats with type 1 diabetes, possibly through its effect of inhibiting Rho kinase activity and thus reducing epithelium-mesenchymal transdifferentiation (EMT). This may guide further animal studies, clinical trials on the importance of benidipine in diabetic EMT development especially in type 1 diabetic, and the possible mechanism involved. From the long run, it may direct the clinical use of benidipine in treating patients with diabetic nephropathy especially those in T1DM.

## Figures and Tables

**Table 1:** Primer sequences for ROCK1 and *β*-actin.

Primers	Primer sequences	Product	Denature *T*
Rock1	Forward: AAGAGAGTGATATTGAGCAGTTGCG	192 bp	61°C
	Reverse: TTCCTCTATTTGGTACAGAAAGCCA		

*β*-Actin	Forward: AAGATGACCCAGATCATGTTTGAG	146 bp	60°C
	Reverse: TAGATGGGCACAGTGTGGGTG		

**Table 2:** The changes of UTP/24 h, urine NAG activity, Ccr, and Scr.

Group	*n*	Urine NAG (u/L)	UTP/24 h (mg/24 h)	Scr (*µ*mol/L)	Ccr (mL/min)
N	8	14.86 ± 4.79	5.62 ± 2.38	59.17 ± 7.87	4.92 ± 1.03
D	9	26.48 ± 6.49^★^	27.38 ± 7.13^★^	69.08 ± 5.03^★^	2.49 ± 0.66^★^
F	9	19.39 ± 3.57^△^	20.43 ± 4.69^△^	60.93 ± 7.76^#^	3.05 ± 0.77^★^
B	8	21.08 ± 5.26^△^	22.47 ± 3.1^△^	61.24 ± 6.35^#^	2.96 ± 0.83^★^

Compared with N group, ^★^ *P* < 0.01, compared with D group, ^△^ *P* < 0.01; ^#^ *P* < 0.05.

**Table 3:** The changes of blood pressure and glucose.

Group	*n*	BP (mmHg)	Blood glucose (mmol/L)
N	8	86.37 ± 11.24	6.37 ± 0.85
D	9	87.72 ± 10.03	27.84 ± 4.56^★^
B	9	85.26 ± 12.45	28.91 ± 5.39
F	8	86.95 ± 10.27	26.91 ± 3.37

Compared with N group, ^★^ *P* < 0.01.

**Table 4:** The protein expressions of p-MYPT1, ROCK1, *α*-SMA, and E-cadherin.

Group	*n*	p-MYPT1	ROCK1	*α*-SMA	E-Cadherin
N	8	0.87 (0.72~0.95)	0.39 (0.23~0.55)	0.11 (0.06~0.18)	0.95 ± 0.27
D	9	1.32 (0.93~1.62)^★^	0.93 (0.64~1.28)^★^	0.68 (0.57~0.82)^★^	0.25 ± 0.09^★^
F	9	0.86 (0.73~1.25)^△^	0.62 (0.51~0.8)^△^	0.14 (0.08~0.19)^△^	0.45 ± 0.11^#^
B	8	0.93 (0.74~1.39)^△^	0.61 (0.52~0.9)^△^	0.13 (0.08~0.32)^△^	0.44 ± 0.10^#^

*F* value		7.37	20.94	125.26	28.15
*P* value		<0.01	<0.01	<0.01	<0.05

Note: compared with N group, ^★^ *P* < 0.01; compared with D group, ^△^ *P* < 0.01, ^#^ *P* < 0.05.
